# Spatial Rule-Based Modeling: A Method and Its Application to the Human Mitotic Kinetochore

**DOI:** 10.3390/cells2030506

**Published:** 2013-07-02

**Authors:** Bashar Ibrahim, Richard Henze, Gerd Gruenert, Matthew Egbert, Jan Huwald, Peter Dittrich

**Affiliations:** 1 Bio Systems Analysis Group, Institute of Computer Science, Jena Centre for Bioinformatics and Friedrich Schiller University Jena, Ernst-Abbe-Platz 2, D-0007743 Jena, Germany;E-Mails: richard.henze@uni-jena.de (R.H.); gerd.gruenert@uni-jena.de (G.G.); matthew.egbert@gmail.com (M.E.); ja.hu.03@uni-jena.de (J.H.); 2 German Cancer Research Centre, DKFZ-ZMBH Alliance, Im Neuenheimer Feld 280, 69120 Heidelberg, Germany

**Keywords:** keyword, rule-based, modeling, simulation, 3D space, SRSim software, kinetochore structure, spindle assembly checkpoint, mitosis, structural analysis

## Abstract

A common problem in the analysis of biological systems is the combinatorial explosion that emerges from the complexity of multi-protein assemblies. Conventional formalisms, like differential equations, Boolean networks and Bayesian networks, are unsuitable for dealing with the combinatorial explosion, because they are designed for a restricted state space with fixed dimensionality. To overcome this problem, the rule-based modeling language, BioNetGen, and the spatial extension, SRSim, have been developed. Here, we describe how to apply rule-based modeling to integrate experimental data from different sources into a single spatial simulation model and how to analyze the output of that model. The starting point for this approach can be a combination of molecular interaction data, reaction network data, proximities, binding and diffusion kinetics and molecular geometries at different levels of detail. We describe the technique and then use it to construct a model of the human mitotic inner and outer kinetochore, including the spindle assembly checkpoint signaling pathway. This allows us to demonstrate the utility of the procedure, show how a novel perspective for understanding such complex systems becomes accessible and elaborate on challenges that arise in the formulation, simulation and analysis of spatial rule-based models.

## 1. Introduction

Large molecular complexes, like the kinetochore [1] or the spindle pole body (SPB, yeast centrosome) [2], consist of many different proteins and other components. Due to the combinatorics of modification and cluster formation, a detailed reaction network model of the system would need to handle a prohibitively large number of molecular species [3]. The number of molecular species can easily go into the millions and can be virtually infinite. Time and space add further dimensions to the combinatorial complexity [4]. Conventional modeling approaches, such as differential equations, Boolean networks and Bayesian networks, are insufficient to cope with combinatorial explosion, due to their use of a restricted state space with fixed dimensionality [5]. To overcome this problem of combinatorial explosion, several rule-based modeling approaches have been developed [6–11], which allow one to define a reaction network implicitly [12]. Extending this line of research, we have recently presented SRSim [13], which combines the BioNetGen language (BNGL) for rule-based reaction systems [6–11] with a three-dimensional coarse-grained simulation building upon the LAMMPSmolecular dynamics (MD) simulator [14]. SRSim fills a gap located in between the fine-grained MD simulation models, which do not allow for the formulation of reaction networks, and 2-D or 3-D graph drawing software tools, which do not include any possibility for dynamic simulation.

Here, we describe (i) the work flow for how to construct a rule-based spatial model using SRSim [13]; (ii) how to integrate experimental data from different sources; and (iii) how to analyze the output of that model. We demonstrate the method by constructing a model of the human mitotic kinetochore, which is a multi-protein structure that assembles on centromeric DNA through complex pathways coordinated by cell-cycle phases [15]. Kinetochores play a central role in mediating chromosome segregation during mitosis [15]. They are of particular interest, because malfunction can cause aneuploidy and cancer [16–21]. Understanding the function and the structure of kinetochores is challenging, both experimentally (due to their complex dynamical structure) and theoretically (due to the combinatorial explosion in the number of intermediate complexes).

A kinetochore consists of a large number of different, interacting proteins, each possessing various sites for binding to other proteins or for modification, e.g., by phosphorylation. To overcome the difficulties of modeling such heterogeneous and complex systems, rule-based modeling relies on implicit descriptions for the binding and modification reactions, where “meta” rules are listed instead of writing all possible reactions. Each meta rule usually describes a large or infinite set of possible concrete reactions. Consider, as an example, a simple polymerization, where a monomer, *A*, can form polymers, *AAandAAA*,… via reactions, like *A* + *A* → *AA*, *AA* + *A* → *AAA*, *etc.* The resulting reaction network possesses an infinite number of molecular species and reactions, thus it cannot be described by conventional frameworks, like the Systems Biology Markup Language (SBML). The same system can be described by the definition of an elementary molecule (monomer; compare Table 1 for more details about the terminology), *A*(*a*, *a*), possessing two binding sites, named “*a*”, and one “meta” rule of the form *A*(*a*)+*A*(*a*) → *A*(*a*!1).*A*(*a*!1), which defines the formation of a bond, named “1”, between binding sites, named “*a*”. Note that the rule does not care whether the second binding site of an elementary molecule, *A*, is occupied. To specify those rules, our SRSim uses a formal language compatible with BNGL [6,8], though alternative languages, like kappa [22], would have been equally suitable.

**Table 1.** Terminology box.

**Term**	**Specification**
*elementary molecules*	Basic building block, consisting of a set of sites. Here, in our example, representing a protein, like Cdc20, or a complex, like anaphase promoting complex (APC) or CenpC
complex molecule	A connected set of *elementary molecules*
site	Region of a *elementary molecule* where specific other *elementary molecules* can attach.
reaction	A transformation of a (possibly connected) set of elementary molecules into a modified (possibly differently connected) set of elementary molecules.
rule	An implicit definition of a family of reactions, specified by the common features of the involved *elementary molecules*. For complex molecules, only specific binding sites are explicitly described and others are omitted
parameter set	Values defining the simulation and model. Changing a single value will lead to a new parameter set.

A second fundamental challenge is that spatial dynamics are important to the organization of kinetochores, which is a key element in understanding their function, e.g., with respect to the spindle assembly checkpoint [23]. Note that spatial properties, like molecular geometry and location, add further dimensions to the combinatorial complexity. In order to cope with this problem, we have presented a novel spatial and rule-based simulation (SRSim, Gruenert *et al.* [13]), which extends a rule-based framework by adding information about the molecules' geometry and which allows spatial stochastic simulations, based on the molecular dynamics software, LAMMPS [14]. Note that our approach is different from rule-based spatial simulations using partial differential equations or spatial versions of the Gillespie algorithm. In particular, molecules in our model possess a volume and orientation, which generates additional constraints on the applicability of reaction rules that are not present in the aforementioned spatial simulation methods. For example, a particular definition of molecular size and bond angles will prevent the formation of small ring-like molecules in the polymer example above. Another interesting feature is that transport processes can emerge, which would not appear in a (stochastic) partial differential equation (PDE) [13]. The software and its source code are freely available online [24].

In the next section, we present the process of constructing a spatial rule-based model, describing the information necessary for the construction and how to analyze the simulation output. Then, in Section 3, we demonstrate the application of the method on a model of the human kinetochore.

## 2. Methods

**Figure 1.**
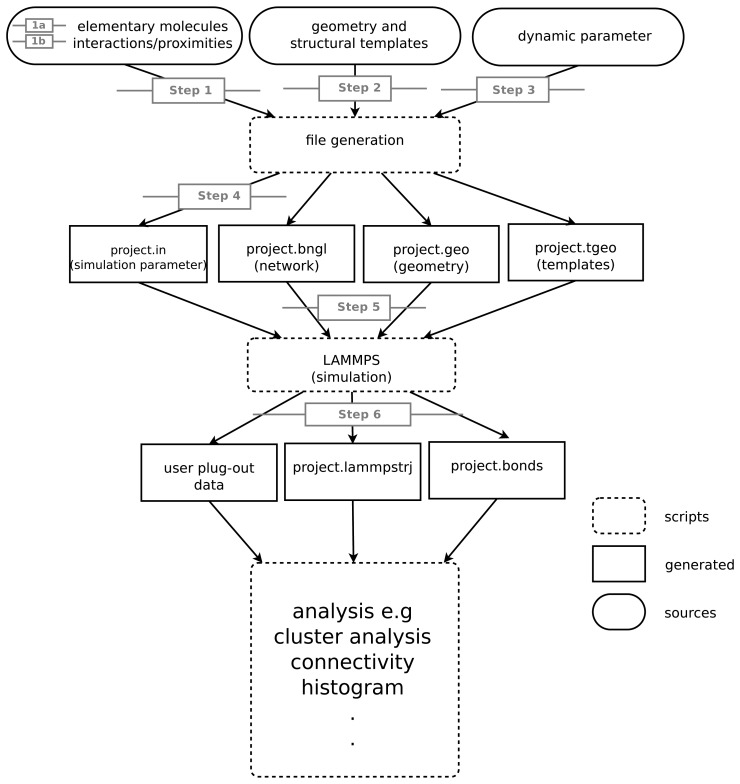
A flowchart depicts the steps and processes necessary for creating a spatial rule-based simulation. First, we prepare three tables with the geometry of the elementary molecules, their interactions and the structure templates (if any). These tables are the input for the file generator that will then translate the geometry information and rules in the SRSim language and will generate several files (namely, .in, .bngl, .geo and .tgeo). The final step is to run the simulation and to store the user defined output, which is the foundation for the analysis. The grey marks correspond to the steps mentioned in Section 2.

The model we construct consists of an implicit description of a reaction network and a description of the geometry of the *elementary molecules*, forming the molecules of the network. Furthermore, it is necessary to specify the dynamics for the simulation (summary Table 2). We suggest the following procedure for building the model and simulation. In the following sections and in Figure 1, we will elaborate on the steps given here.

**Table 2.** Overview of the required model specifications and typical experiments for obtaining them. FCS, fluorescence correlation spectroscopy; FCCS, fluorescence cross-correlation spectroscopy; FRET, Förster resonance energy transfer; FRAP, fluorescence recovery after photobleaching.

**Model parameter/specification**	**Typical experimental procedure**
List of elementary molecules	Dependent on model assumptions and no specific experimental procedure
List of interaction rules	Yeast two-hybrid (Y2H), mammalian two hybrid (M2H), co-immunoprecipitation, tandem affinity purification, FCS, FCCS, FRET (see Section 2.4 for details)
Geometry of *elementary molecules*	Estimates based on molecular mass (see Section 2.3), or protein structure methods (X-ray crystallography [25,26], NMR spectroscopy [27,28], and Dual polarization interferometry [29])
Diffusion coefficient (*D*)	FCS, FCCS, and FRAP
Dissociation rate *k_off_*	FRAP, and FCS
Association *k_on_*	Based on *k_off_* and the relative concentration

Step 1: Construct the reaction network.
  Step 1a: List the basic *elementary molecules* (e.g., proteins).
  Step 1b: List the interaction rules (e.g., complex formation).
Step 2: Specify the geometry of *elementary molecules*. This may include predefined *structural templates* for *elementary molecules* with non-spherical shapes.
Step 3: Obtain dynamical parameters (e.g., for binding).
Step 4: Specify simulation parameters (e.g., volume and simulated time).
Step 5: Specify simulation output modes for a later analysis and visualization.
Step 6: Perform simulation and analyze simulation output.

### 2.1. How to List the Basic Elementary Molecule (Step 1a)

**Figure 2.**
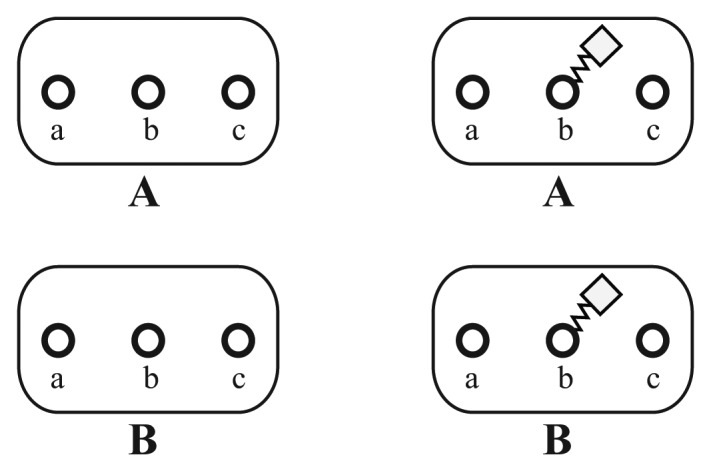
Examples of typical *elementary molecules*. On the left side, two different *elementary molecules*, *A* and *B*, are displayed with three *sites*, *a*, *b* and *c*, which are represented by the circles. The attached squares on the right symbolize a modification of the *site*, *b*. In this example, no further information about the kind of modification is given.

As a first step, the basic reacting particles, called *elementary molecules*, should be defined. In our example, we define for each involved protein one *elementary molecule*. In general, *elementary molecules* can also represent arbitrary types of components, like atoms, small molecules, protein complexes or DNA. In Figure 2, two different *elementary molecules*, *A* and *B*, are shown. The circle symbols, *a*, *b* and *c*, denote *sites*. A modification of site *b* is introduced to the displayed molecules. All *sites* are unaffected on the left, while *b* has been modified unspecifically on the right.

An example of complete BNGL source files can be found in Supplementary Materials 5.

**Figure 3.**
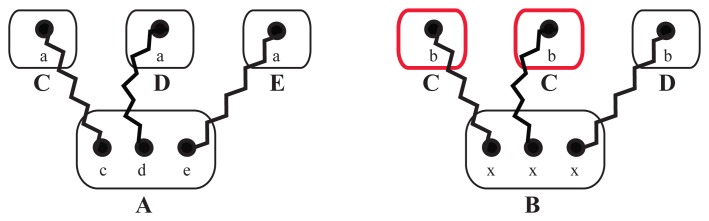
Illustration of binding site specifications with generic or with specific binding sites. On the left, molecule *A* has three specific *sites*, *c*, *d* and *e*. The *elementary molecules*, *C*, *D* and *E*, are connected to molecule *A* by using the appropriate binding *site* of the lower case name only—site *a* is suitable for molecule *A* only Those specific binding *sites* do not allow the link between *A* and *C* twice, due to the fact that only one *site c* exists. Molecule *B*, on the right, in contrast, has three times the same *site*, *x*. Here, the underlying rules would allow any molecule from {*C*, *D*, *E*} to bind to any binding site, *x*, reducing the number of necessary rules to describe the system. As a result, it would not be guaranteed that the molecules binding to *B* are distinct, as exemplified by two molecules, *C*, and one molecule, *D*, binding to *B*.

For each *elementary molecule*, we also have to define a set of *sites* that can later be bound to other *elementary molecules* or can be modified, e.g., phosphorylated. Furthermore, as shown in Section 2.3, each binding site can be arranged in particular angles to define the interaction geometry. Binding sites of a *elementary molecule* can be given the same name, so that reaction rules do not distinguish between them. This might simplify the set of necessary reaction rules, but sacrifice specificity. If we have no further informationabout structure, we suggest the following two alternatives: Case 1: We define the *elementary molecules*, {*C*, *D*, *E*}, that should bind to *A*; according to our data, one specific binding site in *A* (Figure 3, left). In this case, each of the molecules, {*C*, *D*, *E*}, can bind only once to *A* and all {*C*, *D*, *E*} can bind simultaneously. Case 2: We define *n* generic binding sites having the same name (Figure 3, right). In this case, different combinations of up to *n* molecules from the set {*C*, *D*, *E*} can bind to *A*. This approach does not allow one to define specific binding angles for a particular binding partner, *D*, in Step 2, because different binding partners, and thus, their angles, cannot be distinguished any more.

### 2.2. How to List the Interaction Rules (Step 1b)

**Figure 4.**
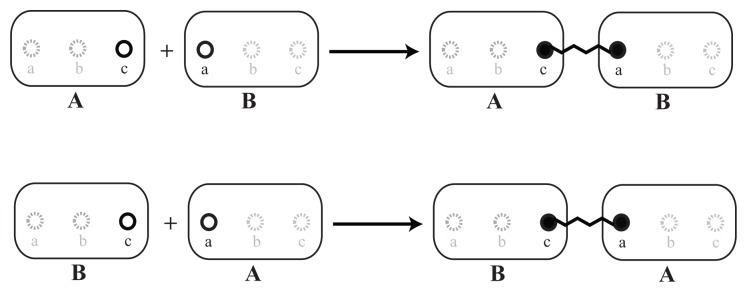
Examples of implicit reaction rules. Illustrative presentation of different rules between two molecules. Both displayed rules describe an interaction between the *elementary molecules*, *A* and *B*. The difference is in the participating *sites*. This example could lead to a double connection between *A* and *B* if there were no steric restraints. Another possibility is the formation of polymers of indefinite length. Please note that the binding sites in grey are not part of the rule, *i.e.*, the bond and modification state of the grey sites is not considered for the rule to be applicable. All that is required for the upper rule to fire is that a molecule, *A*, has a free binding site, *c*, and that a molecule, *B*, has a free binding site, *a*. Then, these free binding sites are bound together. Via the grey binding sites, large molecule graphs could be connected, which would not interfere with the reaction.

Experiments that deliver information on binding and proximities can be mapped to reaction rules that bind two *elementary molecules* together. In this step, the information on how these elements interact needs to be specified: Using the *elementary molecules*, the reaction system can be described via an implicit reaction rule, as displayed in Figure 4. Here, an example is shown, which represents the interaction of the *elementary molecules*, *A* and *B*, from Figure 2. The greyed out *sites* of the molecules, *A* and *B*, do not have to be specified. Omitting them means that they can be in either a bound or an unbound state or modified in any way. The solid *sites*, *a* and *c*, in contrast, have to be in the specified state on the left-hand side of the reaction rule, that is, they have to be unbound before the reaction can happen.

**Figure 5.**
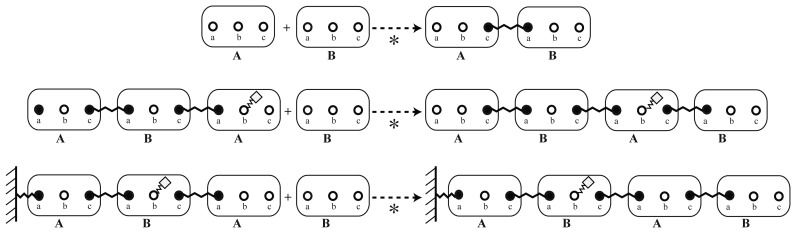
Instances of the reaction rule. Explicit instances of the implicit reaction rule defined in Figure 4 (upper rule), whereby, * refers to the application of the implicit rules. As described for Figure 4, the *sites* that were not mentioned (grey) by the rule can be modified or bound to other molecules.

To illustrate the concept of an implicitly defined reaction system, different instances of the polymerization rule (Figure 4) are displayed in Figure 5.

In this example, the modification at *site b* is of no importance for the polymerization dynamics. Furthermore, the reaction rule does not distinguish between molecules that are already bound to some other structure and between molecules that would assemble freely from two unbound monomers.

Note that a bond in the model does not necessarily need to represent an actual chemical bond. Assume that we have only proximity data (e.g., Förster resonance energy transfer (FRET) data), which indicates that two molecules are close to each other. It could make sense to define a binding reaction for these two molecules. In this case, the bond in the model should be interpreted as an unknown mechanism bringing these two molecules into close proximity.

#### How to Specify Context-Dependent Binding Rules

It is possible to specify context for the reaction rules to customize the binding dynamics. For example, the system under consideration could exclude the assembly of new structures in the absence of a nucleation structure (like the *γ*-tubulin in the case of microtubule formation [30–32]). Such a behavior can easily be incorporated into the rule-based model by a slight modification of the polymerization rule.

**Figure 6.**
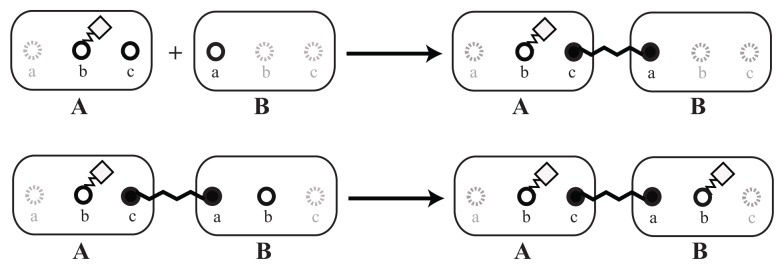
Context-dependent reaction rules that exclude the assembly in the absence of a nucleation structure. That is, polymerization will only happen when a protein modification at site *b* is already present. When a non-modified protein is bound to a modified one, the modification is passed on by the second rule. These new rules would have to replace the reaction rules defined in Figure 4 to avoid the context-free assembly reaction to happen, as well.

For example, we can add a dependency on a modification of *site b* that will denote, if the molecule is already in some way connected to the nucleation structure. Furthermore, we will add one more rule that will propagate the modification of site *b* to adjacent molecules, as shown in Figure 6.

### 2.3. How to Specify the Geometry of the Elementary Molecule (Step 2)

For the spatial rule-based simulation, the geometries of the participating particles need to be defined in addition to the diffusion coefficient (cf. Section 2.4). In the simplest case, a spheric shape is assumed for every molecule in the reactor. Nonetheless, for any particle, a more complex shape can be assembled from a collection of spheric *elementary molecules*, as shown in the next section.

To model the spheric building blocks, the following values are required: the *elementary molecule*'s mass, *m*, its diameter, *d*, and the geometry of its *sites*. When the size is unknown, the mass of the molecules can be used for a coarse approximation of its volume, *V*, and, thus, of its diameter, *d*, by assuming the same density, *ρ*, for all particles:

(1) ρ=m/V

$$d=2\sqrt[{3}]{\frac{4m}{3\rho \pi }}$$

**Figure 7.**
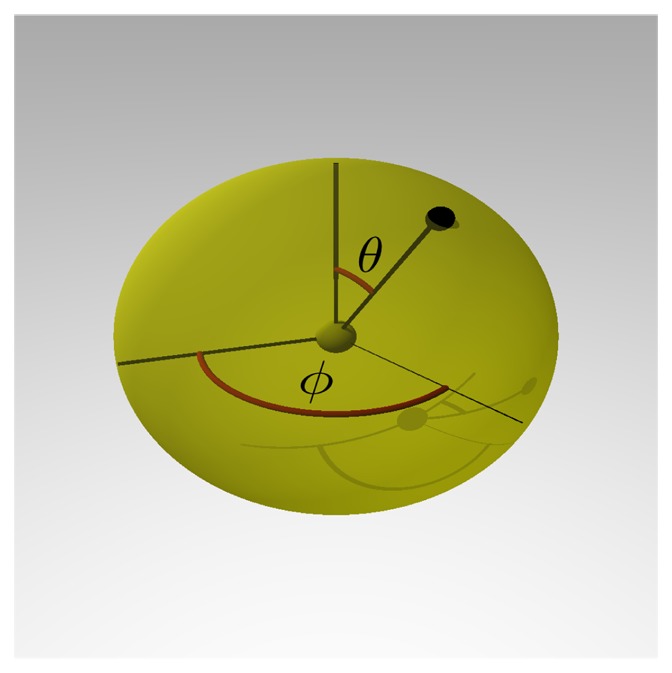
Specification of binding sites. Exemplary specification of a binding site with the angles, *φ* and *θ*, and the length of the bond, *r*. These variables correspond to spheric coordinates. Coarsely comparable to geographic coordinates, (90° − *φ*) would correspond to the latitude, *θ* to the longitude and *r* would be the distance between the location and the Earth's center. While *φ* gives the angle between the binding site and the vertical zenith axis, *θ* gives the rotation around this zenith axis.

For the interaction with reaction partners, the geometry of all the *elementary molecule*'s *sites* is then specified in polar coordinates, *i.e.*, by the two angles, *φ* and *θ*, and the length of the binding *site* (compare Figure 7). This length is typically the radius of the *elementary molecule*, which means reactions occur on its surface. Next to the binding angle, there is also the possibility to define dihedral angles. While this information can be specified if (partially) present, in many cases, no data on the structure or on binding angles will be present, so that the whole surface of the spheric particles will have to be considered equally reactive.

#### How to Predefine Structural Templates

**Figure 8.**
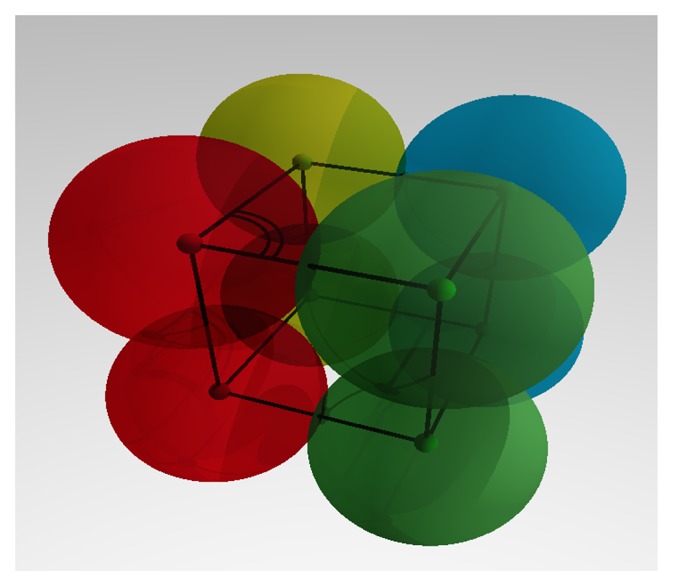
Structural template. Example of a structural template, showing a cube shaped assembly composed of eight *elementary molecules*. As a rigid body, this structure will keep its shape during the whole length of the simulation.

If there exists detailed information about the shape and structure of a molecule or complex, a structural template can be defined. It can consist of multiple sphere shaped *elementary molecules*, assembled in a particular shape. Now, initial coordinates for each of the *elementary molecules* can be specified. The relative coordinates can be fixed, so that the whole template behaves as a rigid particle or, at least, starts from a defined spatial configuration. For example, to approximate a cube shaped complex molecule, the position of the eight edges in space could be specified for eight equidistant connected elementary molecules with fixed angles of 90 degree, as displayed in Figure 8. The edges of the cube refer to the *sites*, while the illustrated angle is defined between three *elementary molecules*, so the whole template becomes rigid if all those angles are fixed.

### 2.4. How to Obtain Dynamical Parameters from Experiment (Step 3)

While the reaction system described so far can be analyzed algebraically [33], a dynamic simulation requires the following kinds of parameters: First order reaction rate constants for dissociation and modification reactions, as well as second order rate constants for binding and modification reactions. Furthermore, for spatial simulations with explicit diffusion of particles, diffusion rates should be known. We now present typical measurement techniques for determining these values.

For obtaining diffusion, association and dissociation rates and relative concentrations, many methods exist. Most of these are based on fluorescence microscopy, which provides an efficient tool to study molecular processes in either fixed and living cells [34]. Both fluorescence correlation spectroscopy (FCS) and two-color fluorescence cross-correlation spectroscopy (FCCS) measurements provide information about diffusion, protein-protein interactions and average concentrations. These measurements can be conducted either *in vitro* or *in vivo* [35] (for a review, see [36,37]). More elaborate techniques, including fluorescence recovery after photobleaching (FRAP), Förster resonance energy transfer (FRET), fluorescence localization after photobleaching (FLAP) and fluorescence lifetime imaging microscopy (FLIM), exist. These techniques provide either protein diffusion and/or binding characteristic between proteins (herein, only the dissociation rate, *k_off_*, can be obtained). For a review, we refer to [38]. In addition to those biophysical techniques, there are biochemical methods that are more specific for interactions among proteins, like yeast two-hybrid [39], mammalian two hybrid (M2H) [40], co-immunoprecipitation [41–43] and tandem affinity purification [44]. In addition to those experimental techniques listed above, other *in-silico* interaction prediction methods are available, such as docking [45] or co-evolution [46]. Furthermore, interaction databases, like the STRING database [47,48], are available.

Each of these techniques has their own advantages and disadvantages. In order to make meaningful and accurate quantification out of experimental data, the right models and methods should be carefully chosen. A very basic example is that, while using FRAP, you can calculate the off rate, *k_off_*. The naive approach of fitting the data to single or double exponential functions does not reflect the physical reality. That is because fitting data to a first order reaction, while the correct reaction is of the second order, leads to unit inconsistencies (the forward reaction rate for the first order is *sec*^–1^ and for the second order reaction is *sec*^–1^*Mol*^–1^). This is particularly important, since *k_off_* is essential to further calculate your *k_on_*, if relative concentrations of the related proteins are known. Thus, for a complex or unknown mechanism, a detailed mathematical model is required (we refer, for example, to the successful FRAP model applied to PMLnuclear bodies [49]).

Moreover, it is important to carefully interpret the means of signaling output for these techniques. For example, the FRET signal observable range of distances is limited [50–52]. The donor and acceptor fluorophores should be in close proximity to each other (around 10 nm or 14 nm), otherwise, no FRET signal can be detected [53]. Nonetheless, a weak or missing FRET signal does not imply large distances.

### 2.5. How to Specify the Simulation Parameters (Step 4)

For a spatial particle simulation, in addition to the description of the reaction dynamics, further parameters for the reaction vessel, as well as a simulation protocol can be specified; in particular, the size and shape of the reactor, its boundary conditions, the initial state of the reactor and the length of the time-steps (Table 3).

A thermostat for the Brownian motion of particles and other forces that constrain the behavior of all particles or of subsets can dynamically be adjusted. Doing this in a fixed time regime leads to the definition of a simulation protocol, e.g., first equilibrate the simulation system for 10^5^ time-steps, then add additional molecules of type X, then equilibrate for another 10^5^ time-steps, then add a force from an electric field on some of the particles and, finally, simulate for 10^6^ time-steps. For more details on the possible configuration and simulation protocol options, we refer to [13,54].

**Table 3.** Main simulation parameters.

**Name**	**Example values in Section 3**	**Meaning**
Reactor volume	800Å × 400Å × 600Å	Size of the reaction space
Reactor Shape	Cubic	Shape of the reaction space
Boundary conditions	Reflective	Boundary conditions can be reflective, harmonic or gran, for example
Initial states	Random or fixed	The initial state can be specified by exactly specifying components and their initial positions. Alternatively, the positions are generated randomly.
Simulation time	8 × 10^6^ time-step	Duration of the simulation
Force	Leonard-Jones	The conservative force between particles additional to the friction. Other possibilities are EAM, hybrid LJor no interaction.

### 2.6. How to Specify Simulation Output for a Later Analysis and Visualization (Step 5)

**Figure 9.**
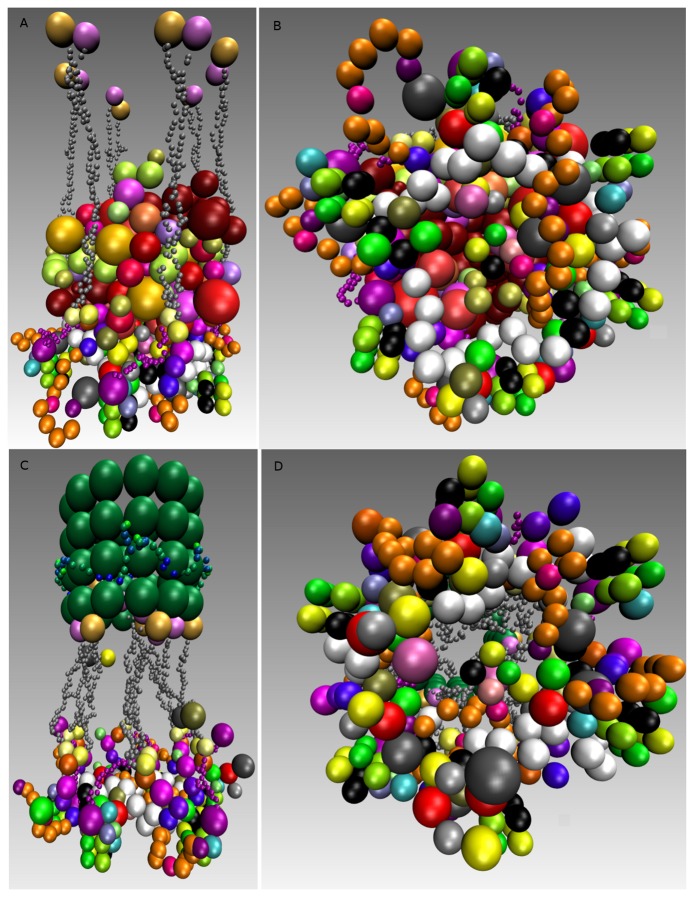
Single rosette-like structure assembly in mitosis. (**A**) Shows a rosette-like structure where SACis active and no microtubule is present or attached; (**B**) Same as in Panel A, showing the rosette-shape in top view; (**C**) Shows a rosette-like structure attached to a microtubule and including the Ska complex, where the Spindle Assembly Checkpoint (SAC) switched off; (**D**) Same as Panel A, showing the rosette-shape in top view similar to Panel B, while SAC is inactive. The distance between two nucleosomes is 14 *nm*, and consequently, the diameter of the rosette-shape and microtubule is 25 *nm*. The rosette-structure and the Ska-complex are 60 *nm* apart. The attached, as well as the unattached kinetochore took about one hour to simulate.

During and after the simulation, it is possible to write various information to files. Typically, snapshots of the position of each particle are written regularly, for later structural analysis and visualization, as well as for tracking and analyzing the simulation process. These datasets can become quite big, so that there is a trade-off for the experimenter between saving disk space and the temporal resolution of the simulation trajectories. For a first impression of the reactor contents, we used the Visual Molecular Dynamics software (VMD) [55]. It produces plots of the particles in the reactor along the dumped simulation snapshots, leading to visualizations, as shown in Figure 9.

Further useful information is in which bonds are formed or broken during the simulation process at which time. Especially if not focusing on the 3D structure, but on the bond-network, the graphviz package produces a visualization of this network of actually realized interactions. Next to the already implemented general methods to this aim, there are simulator modules for the output that can be modified for special needs. For example, in the case of the kinetochore, more specialized information can be extracted by automatically determining if a path along connected molecules exists from one nucleosome to another.

### 2.7. How to Analyze Simulation Output (Step 6)

The raw result of each simulation is the position of each *elementary molecule* in space, varied over time. The particles' trajectories in the simulator are influenced by the chaotic interactions between the *elementary molecules*, their stochastic reactions, as well as by the random forces implementing Brownian motion. Additionally, every *elementary molecule* starts the simulation at a random position in the reactor, influencing the position of the molecules at any later time-step. Hence, each simulation instance, even from a single model, will produce different trajectories. They might differ in small details only or even vary in macroscopic or structural properties of the assembled complexes. Nonetheless, some final reactor states will be more probable than others, e.g., the formation of a correctly assembled kinetochore complex. Therefore, we are interested in the spatial probability distribution of all the constituting particles, and therefore, sample the space of all possible final configurations by running multiple simulations with varying random seeds.

However, also for different parameter sets, several simulations are run to discover all possible structures. To compare a hypothesis against a null hypothesis, two or more parameter sets are simulated. This amounts to a lot of simulation data, which is computationally and analytically challenging (see the analysis of the inner kinetochore structure [56]). The model presented in Section 3 generated 230 GB of trajectory data and took 3 days to analyze on a compute cluster with 20 state-of-the-art CPU cores.

A number of methods are available to analyze the raw data (Table 4). Typically, a collection of them has to be used to draw up meaningful conclusions. A key question to answer is if two conformations of a cloud of *elementary molecules* in space represent the same structure or not. In addition, one wants to know how dissimilar two structures are. Beyond structure analysis, one can ask, for example, which *elementary molecules* form bonds during simulation or consider the time it took for structure formation and dissolution.

**Table 4.** Analysis methods and required simulation outputs.

**Analysis**	**Simulation requirements**
Measure similarities between two structures: RMSD, Paired RMSD and Local RMSD	Position of each *elementary molecule* for two simulations
A dendrogram for discovering structural diversity within one parameter set	Particle position for multiple simulations of *one* parameter set
A dendrogram for discovering structural diversity between two parameter sets	Particle position for multiple simulations of *multiple* parameter sets
Discover irrelevant reactions	Frequency of realized bounds averaged over several simulations

All these analysis methods can be extended to the dimension of time.

Here, we present—with modifications—our protocol of the structural analysis method used previously [56]. To measure structural similarity, it relies on the root mean square deviation (RMSD) between a chosen set of *elementary molecules*—typically all. Three different RMSDs can be generated: difference of positions, of distances and of local distances. In the rest of the section, *p_i_* and *q_i_*, denote the position of *elementary molecule i* in two different simulation runs that are to be compared, and ‖*x*‖ denotes the Euclidean norm of *x*.

In very simple cases, one can consider the RMSD of the positions of the *n elementary molecules* [57]:

(3) $${\text{RMSD}}_{pq}=\sqrt{\frac{1}{n}\sum_{i=1}^{n}{\left\|p_{i}-q_{i}\right\|}^{2}}$$

As molecules can freely rotate and translate in the simulated space, the *elementary molecule* coordinates require transformation before this method is applied: Translational degrees of freedom (DoF) are eliminated by assuming that one *elementary molecule* is fixed in space (e.g., *p*_0_ = *q*_0_ = 0⃗. Rotational DoF are eliminated by aligning the straight line between two *elementary molecule* pairs (e.g., $\bar{p_{0}p_{i}}\|\bar{q_{0}q_{i}}$ for *i* = 1,2). This scheme can only be used to discriminate small, rigid structures: negligible changes to the macrostructure usually have a larger RMSD than significant changes to a few *elementary molecules*, because the measure relies entirely on the absolute *elementary molecule* position.

To overcome this limitation, we need to incorporate relative position information. We thus consider the RMSD of the pairwise distance between *elementary molecules* [56]:

$${\text{paired RMSD}}_{pq}=\sqrt{\frac{2}{n^{2}+n}\sum_{i=1}^{n}\sum_{j=1}^{i}{\left(\left\|p_{i}-p_{j}\right\|-\left\|q_{i}-q_{j}\right\|\right)}^{2}}$$

It increases if and only if the distance between any particles in structure *p* is different from that in structure *q*. Thus, rotational and translational DoF are irrelevant in paired RMSD. The method is robust against some macrostructural variance (e.g., small rotation of a backbone). We apply paired RMSD for the analysis in Section 3.

If one requires to compare structures with high variability in their macrostructure—for example, long polymers, where the exact shape of the backbone does not matter—one can restrict the paired RMSD to consider only the distances of proximate particles. This can be achieved by a cut-off distance, *C*:

$$\text{local}{\text{RMSD}}_{pq}=\sqrt{\frac{2}{n^{2}+n}\sum_{i=1}^{n}\sum_{j=1}^{i}{\left(\min(C,\left\|p_{i}-p_{j}\right\|)-\min(C,\left\|q_{i}-q_{j}\right\|)\right)}^{2}}$$

A low local RMSD implies that the local structure—bonds and component orientation—is relatively unchanged, whereas the macrostructure might have any shape. The notion of what is considered proximate and what is considered insignificant macrostructure is determined by setting the cut-off distance, *C*, to an appropriate value. Because cut-off distance induced spheres around each particle strongly overlap, local RMSD can be used to detect whether substructures are connected. This does not work in general for RMSD or paired RMSD.

**Figure 10.**
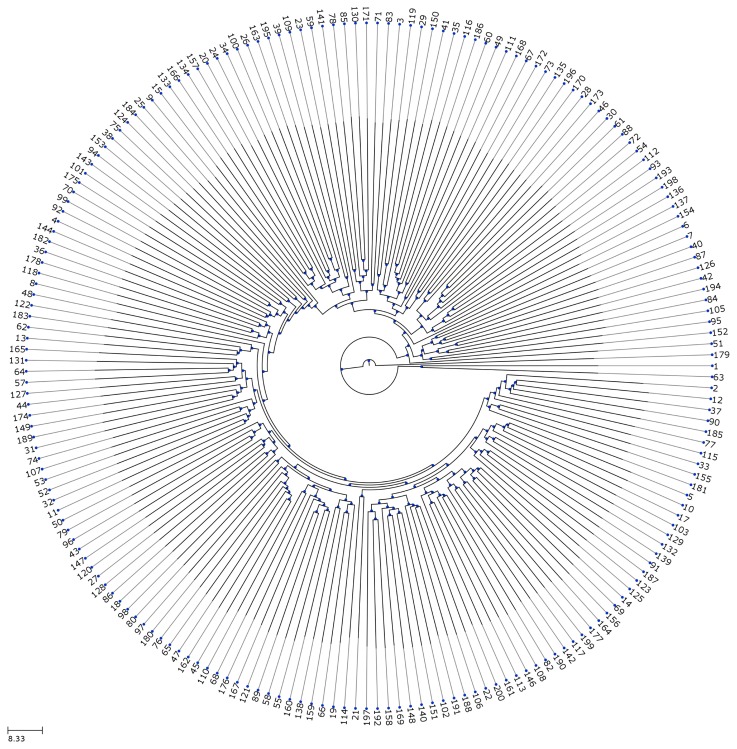
A dendrogram visualizing the distances between 200 structures. Comparison of the structures resulting from 200 simulations by displaying the pairwise root mean square deviation (paired RMSD) using a dendrogram. The leaves are marked with the simulation index. An inner node close to the root denotes high dissimilarity between the branches of the node. The presented tree reveals only inner nodes very close to the root. This leads to the conjecture that all structures are microscopically different, but macroscopically similar. Considering structure 179 as an outlier, this implies that the given model produces exactly one structure.

Each of the three RMSD measures allows one to compute a distance between two structures, *p* and *q*. However, typically, one has to compare *n* ≫ 2 different structures, *p*_1_,…, *p_n_*. Computing the distance for each pair (*p_i_*,*p_j_*) of structures yields a *n* × *n*-matrix of distances. A standard approach to visualize such a distance matrix is a dendrogram [58]. In this tree, each leaf represents one structure from one simulation, and the distance from each inner node to the root is proportional to the negated maximal distance between all structures that are child nodes of this inner node. At a glance, one is able to spot families and similarities between (groups of) structures: the closer to the root the lowest common node between two structures is, the more different they are. Reciprocally, if there are qualitatively different structures in the dataset, the junctions closest to the root distinguish between them. See Figure 10 for an example.

One can use analysis methods that do not rely on the particle positions of each simulation run. One such example is the probability of bond realization averaged over several simulation runs. This can be used to discover if a reactions is superfluous, which, in turn, allows one to derive the smallest set of reactions that explains the formation of a certain structure (model reduction). It can also be used to validate models if an experimentally confirmed bond does not occur in a simulation.

All proposed analysis methods can be extended in the dimension of time. Considering the bond frequency over time allows for several conclusions. A transiently elevated bond frequency hints at a reaction that might be required for structure formation, but not maintenance. A delayed onset of the bond frequency of a mutant compared to a wild-type indicates reactions that are not strictly necessary for a complex to form, but that increase the formation speed. This difference in timing can be critical—for example, during mitosis [59].

## 3. Biological Application: Rule-Based Spatial Model of the Human Mitotic Kinetochore (HMK)

Due to the combinatorial explosion of the number of intermediate complexes, studying the 3D structure of the full kinetochore is challenging, both experimentally and theoretically Conventional modeling approaches, like differential equations, fail to describe the self-assembly process. Recently, a first 3D rule-based model of the inner kinetochore has been proposed based on intracellular proximity between inner kinetochore proteins [56], which suites to cope with combinatorial complexity However, they modeled the S-phase inner kinetochore and used the same geometry for all proteins.

Here, we used a similar approach to study a full mitotic kinetochore model (HMK), including inner and outer kinetochore and the spindle assembly checkpoint modules. We used structural template geometry for CenpC,-T,-Q, Mis 12-, Ndc80-, Mad1:Mad2-, RZZ-, APC- and Ska-complexes and microtubules (Tables 5 and 6). “Rosette”-like nucleosomal shape was suggested for the kinetochore structure in mitosis [15]. We test if this specific structure can be assembled and under which possible conditions. We study several model variants for either attached or unattached kinetochore and with single or multiple microtubules.

**Table 5.** **Molecular geometry information.** We derived the radius of simulation particles from the assumption that molecules are spheric and all have the same density.

**Protein**	**Molecular mass in [kDa]**	**Radius in** [Å]	**Protein**	**Molecular mass in [kDa]**	**Radius in** [Å]
CenpA	16.00	15.33	Apc1	216.50	36.53
CenpB	65.00	24.46	Apc2	94.00	27.66
CenpC	107.00	28.88	Apc3	92.00	27.46
CenpI	87.00	26.95	Apc4	92.00	27.46
CenpK	31.50	19.21	Apc5	85.00	26.75
CenpM	20.00	16.51	Apc6	71.50	25.25
CenpN	39.50	20.71	Apc7	67.00	24.71
CenpO	34.00	19.00	Apc8	69.00	24.95
CenpP	33.00	19.51	Apc10	21.00	16.78
CenpQ	30.50	19.01	Apc11	10.00	13.11
CenpR	20.00	16.51	Apc12	10.00	13.11
CenpS	16.00	15.33	Apc13	8.50	12.41
CenpT	60.50	23.88	Cdc20	55.00	23.13
CenpU	47.50	22.03	Mad1	83.00	26.53
CenpW	10.00	13.10	Mad2	23.50	17.42
CenpX	9.00	12.65	Bub1	122.00	30.17
H3	15.00	15.00	BubR1	120.00	30.00
Dsn1	40.00	20.8	Bub3	37.00	20.27
Mis12	24.00	17.55	Mps1	97.00	27.95
Nnf1	23.50	17.42	Zwint	31.00	19.11
Nsl1	32.00	19.31	Zwilch	67.00	24.71
Hec1	74.00	25.54	Zw10	89.00	27.16
Nuf2	54.00	22.99	Rod1	250.00	38.32
Spc24	22.50	17.17			
Spc25	26.00	18.02			
Knl1	265.00	39.07			

**Table 6.** The inner and outer kinetochore and SAC proteins.

**Protein**	**Interaction partner(s)**
CenpA	CenpB [60], -N [61,62], -C [63,64]
CenpB	CenpW [56], -Q [65] -U [66], -C [67]
CenpC	
CenpI	CenpU [66]
CenpK	CenpO [65], -R [65], -U [65], -N [65]
CenpM	CenpS [56], -U [56], -T [66], H3 [56]
CenpN	
CenpO	CenpP [65], -U [65], -Q [65]
CenpP	CenpU [65], -Q [65]
CenpQ	CenpU [65]
CenpR	CenpU [65]
CenpS	H3 [56], -T[68], -X[69]
CenpT	H3 [56,70]
CenpU	
CenpW	H3 [56,68]
CenpX	
H3	
Dsn1	Mis12 [71], Nsl1 [71]
Nnf1	Mis12 [72], CenpC [64]
Nsl1	Spc24 [72,73], Spc25 [72,73]
Mis12	
Ndc80/Hec1	Nuf2 [74,75]
Nuf2	Spc25 [74,75]
Spc24	Spc25 [75,76], CenpT [77,78]
Spc25	
Knl1	
Cdc20	Mad2 [79–81], BubR1 [82–85], Apc2 [86,87]
Mad2	Mad1 [88–90], Rod1 [91,92], Zw10 [91,92]
Mad1	Bub1 [93], Rod1 [91,92], Zw10 [91,92]
Bub3	Bub1 [94,95], BubR1 [95–97]
BubR1	Bub1 [94,98], Knl1 [99–102], Apc3 [103], Apc4 [104,105], Apc5 [106], Mps1 [107], Rod1 [108], Zw10 [108]
Bub1	Knl1 [100,101]
Mps1	Mis12 [109], Hec1 [71,110]
Zwint	Zw10 [111,112],Knl1 [101], Mis12 [71,113]
Zwilch	Rod1 [114,115], Zw10 [108,111,115]
Zw10	Rod1 [108,115,116], Knl1 [101]
Rod1	

### 3.1. Human Mitotic Kinetochore

Human kinetochores contains over 100 proteins and complexes and can be classified into two functional parts: the inner kinetochore that binds centromeric DNA and the outer kinetochore that binds microtubules. The inner kinetochore is composed of a centromeric CenpA and 16 CCAN (constitutive centromere-associated network) proteins (CenpC, CenpH, CenpI, CenpK to CenpU, CenpW, CenpX) [117]. The inner kinetochore is relatively stable and present during most of the cell cycle [1,118,119], while the outer kinetochore is less stable and forms in mitosis [120,121].

The outer kinetochore proteins and complexes include the KNL-complex (Spcl05/Knl1), Mis12-complex (Dsn1, Nnf1, Nsl1 and Mis12) and the Ndc80-complex (Ndc80/Hec1, Nuf2, Spc24 and Spc25), which are referred to as the KMNnetwork (for a review, we refer to [1]). Additionally, the Ska-complex (Ska1, Ska2 and Ska3/Rama1) is essential for the attachment of the chromosome to the spindle microtubules [122].

Kinetochores are a part of and regulate two crucial mechanisms during mitosis. First, they work as a platform, generating a signal for the Spindle Assembly Checkpoint (SAC, [123]) which mediates the correct attachment between the chromosome and spindle microtubules. Second, they ensure proper tension and orientation of attached chromosomes. Here, we shall focus on both the inner and outer kinetochore together with the SAC pathway.

SAC delays the onset of anaphase, until all chromosomes have made amphitelic tight bipolar attachments to the mitotic spindle. Even one misaligned kinetochore catalyzes the formation of a “wait-anaphase” signal, which then diffuses to counter the ubiquitin ligase anaphase promoting complex (APC) by its co-activator, Cdc20. Activation of APC by Cdc20 triggers chromosome segregation by ubiquitination of securin and cyclin B [124–127]. The core proteins involved in SAC are conserved in all eukaryotes, include MAD (“Mitotic Arrest Deficient”; Mad1, Mad2 and Mad3 (in humans: BubR1)) [128] and BUB (“Budding Uninhibited Benzimidazole”; Bub1 and Bub3) [129]. These proteins work to regulate APC and Cdc20. In addition to these core proteins, the SAC involves several other components. Among these are the Aurora-B [130], the RZZ complex (ROD, Zw10 and Zwilch; for a review, see [131]) and Zwint-1 [112]. The biochemical reactions of SAC activation and maintenance can be thought of in a very simple way as two modules. First is the Mad2-activation and its function in sequestering Cdc20, which is known as the “Template model”. Second is the formation of the MCC complex (which consists of Mad2, Cdc20, Bub3 and BubR1 [126]), which directly binds and inhibits the APC complex (for a review, see [132]). Several conventional detailed models are available in the literature for SAC modules (e.g., [23,59,133–138]). However, none of these models considered molecular geometry and structure. We have recently developed a spatial rule-based model for the S-phase inner kinetochore structure [56]. Here, we present, to the best of our knowledge, the first simplified spatial model of the human mitotic inner and outer kinetochore that also includes the spindle assembly pathway.

### 3.2. The HMK Model

We develop a 3D model of the human mitotic kinetochore (inner and outer) in addition to the spindle assembly checkpoint pathway. This model is based on published data from the literature (interaction, proximity and geometry data). Using a rule-based modeling technique in space, we explore a possible structural kinetochore layout assemble. We apply different model variants, namely, unattached kinetochore, attached kinetochore, single microtubule and multiple microtubules.

### 3.2.1. Model and Simulation Assumptions

**Figure 11.**
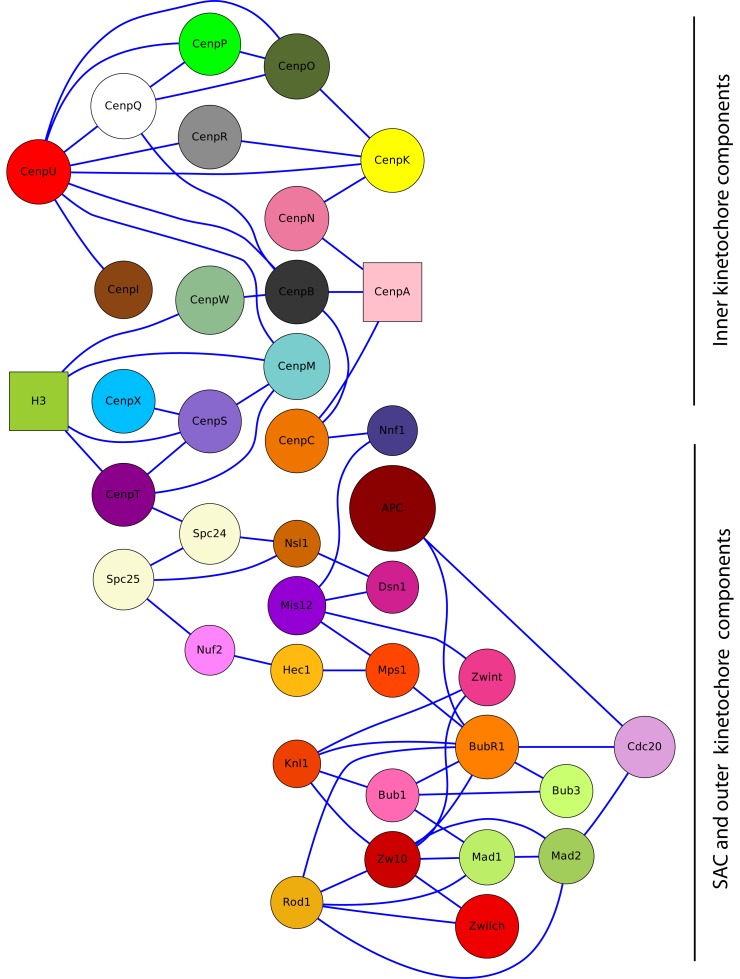
Map of all interactions used for the Human Mitotic Kinetochore (HMK) model (see also Table 6). The nodes in this graph represent a protein or complex, while the edges depict interactions between them. The colors and names of the vertices are the same as in Figures 9 and 12. Square shapes are the two nucleosomes, which are the anchor points for our model calculations. The CenpA nucleosome contains CenpA1, CenpA2, H41, H42, H2A1, H2A2, H2B1 and H2B2. The H3 nucleosome contains H31, H32, H43, H44, H2A3, H2A4, H2B3 and H2B4.

We specify the size of the spheres (or chain of spheres), since proteins have a rather similar amino acid density [139,140]. We adjust the spherical volume of each CCAN protein, due to its molecular mass relative to the density of histone H3 (Table 5). Our coarse-grained simulator allows us to model detailed molecule structures. Therefore, we consider the most known structures as a chain with similar or different parts (e.g., [68,78,141,142]) and all other proteins' structures as spheres, namely, CenpC,-T,-Q, Mis12-, Madl:Mad2-, Ndc80-, RZZ-, APC- and Ska-complexes and microtubules (Tables 5 and 6). The binding domains (listed in Table 6 and shown in Figure 11) are translated into reaction rules (as mentioned in Section 2.2). Because we are not interested in a transient behavior, we start with randomly chosen positions and simulate the system 6 × 10^6^ time-steps and take only the final state for our analysis. After this time, only small changes could be observed. In our model, we simulated the time it needed to form a rough structure twice (the actual structure, but not all *elementary molecules* are bound), which took an average 3 × 10^6^ time-steps).

For unattached kinetochore model variants, we used 25 different molecular species and 10 templates (CenpC,-T,-Q, Mis12-, Ndc80-, Mad1:Mad2-, RZZ- and APC-complexes), as in Table 5 and 6. For the attached kinetochore model variant, we also include two templates for the Ska-complex and microtubule(s), which remove SAC proteins from the kinetochore. They are distributed randomly in the reactor volume: (*x* = (−400Å; 400Å), *y* = (−400Å; 800Å), *z* = (−400Å; 400Å)). At each time-step, all molecules are addressed to move freely according to Brownian motion (we dump the relative position of each particle every 5 × 10^3^ time-step). The two nucleosomes containing either H3 [143] or CenpA [144] are rigid molecules and have fixed positions. Each of the nucleosomes is represented by eight simulation particles that are bound to each other rigidly. If two *elementary molecules*, listed in the reaction table, come close together (depending on the length of their binding site), they bind regarding the defined rule under the conditions of Section 2.2. Initially, only the seven nucleosomes are able to bind anything.

Switching the SAC signaling on and off is controlled by the switching binary parameters (remove some reactions). For instance, in the “Template model”, the formation of Mad1:C-Mad2:O-Mad2* can take place only as long as the kinetochore is unattached [135].

### 3.2.2. Simulation Outcome

We first consider a simple kinetochore, which is not yet attached. In other words, we modeled the inner and the outer kinetochore along with the SAC signaling (checkpoint is active) and no microtubules present. We aim to test if there is a possible way and under which setting a rosette-like structure can be assembled.

In our setting, for the realization of a complete hexagonal rosette-shaped structure, we set the number of every *elementary molecule* and chain-molecule to six, except CenpN and CenpA, which are largely absent from kinetochores during mitosis [61]. We define the reactor size and locate the CenpA containing nucleosome in the middle on a fixed position. The other six H3 containing nucleosomes are placed evenly around the CenpA with a fixed distance of 14 nm. As the exact nucleosomes orientation is unknown, but the position must keep fixed, a rotation around the center of each nucleosome was added, so that it is free to align during the assembly process. All molecules and structural templates (CenpT, -C, -Q, APC complex and MCC complex) undergo Brownian motion in a centering drag-fix (every time-step, a force is added, which causes every particle to be slightly dragged into a sphere around the center of the reactor), which ensured the availability of molecules to ‘react’ and no uniform distribution in the whole reactor.

The simulations result in six bridges, connecting two of the nucleosomes, with many cross-links. We found that the overall rosette-like chromatin structure can be clearly assembled under our HMK model assumptions (see Figure 9A,B and Supplementary Materials Video 1). We went further to see if this structure in principle can solely be formed when the kinetochore is unattached and the SAC mechanism is on. To do this, we used the same model setting as before and added the Ska complex and simplified template for a microtubule (Table 5). We also kept the same assumptions, except the change in the SAC signaling, which is switched off (inactive). In other words, some SAC moduli, like the “Template model”, are switched off once the microtubule is attached. Additionally, microtubule attachment removes the SAC pathway away from the kinetochore. Our simulation clearly shows a well-defined rosette-like structure (see Figure 9C,D and Supplementary Materials Video 2), where the Ska complex is presented in a w-shape dimer, as recently has been proposed [122].

**Figure 12.**
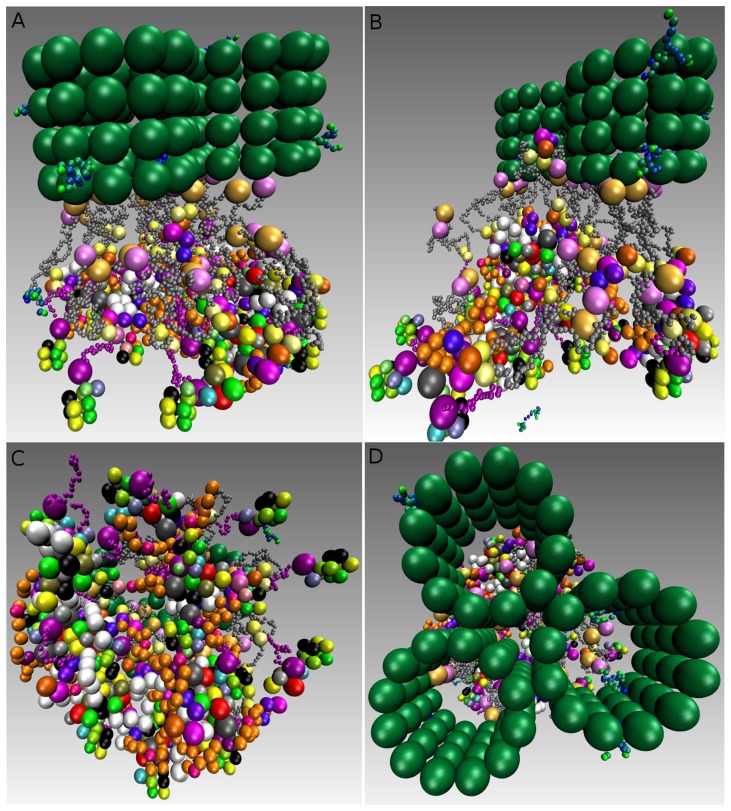
Multiple rosette-like structures assembly in mitosis. (**A**) Shows three rosette-like structures attached to three microtubules; (**B**) Same as in (**A**), where only two microtubules are present and attached with many cross-links occurring; (**C**) Shows that the assembly of the well-defined rosette-structures cannot be obtained for the multiple microtubules attached (**A**); (**D**) Top view of the simulation process shows the arrangement of the microtubules in (**A**). Simulations with multiple microtubules took about three hours.

We asked what happens if we consider three microtubules instead of a single microtubule along with a three-fold increase of protein copies. The simulation resulted in forming obscure rosette-like shapes after all microtubules have attached (Figure 12A,C,D and Supplementary Materials Video 3). This led us to go more thoroughly into analyzing the effect of microtubules. We re-run the simulation, where only two microtubules were available and attached with a three-fold protein amount. Interestingly, in this case, no rosette-shape was formed (Figure 12B and Supplementary Materials Video 4).

Taken together, a rosette-like chromatin structure can be assembled for both an unattached kinetochore and an attached kinetochore to single microtubule. In the case of two or three attached microtubules, we could not observe rosette-structures in simulation. The later rises the possibility that microtubules influence the chromatin-structure after attachments, which may be independent of SAC-component removal.

### 3.2.3. Analyzing the Simulation Output of the HKM Model

In this section, we show how to apply some analysis methods (as described in Section 2.7) to the human mitotic kinetochore structure. ¿From these analyses, we do not aim to draw out a biological conclusion, as there are no empirical data available on mitotic kinetochore structure for comparison. We shall instead raise some speculations that could be of interest for experimentalists to test.

**Figure 13.**
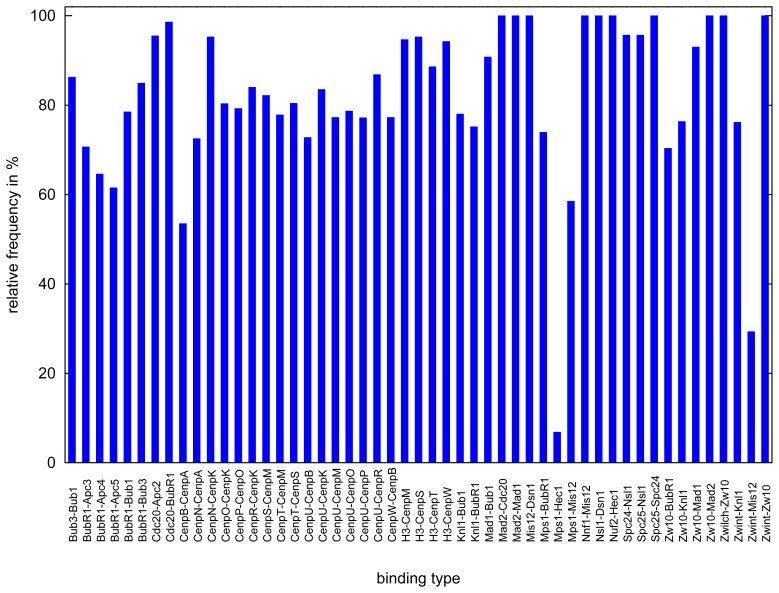
Histogram showing percent of number of possible bindings. We record all realized bonds at the final time-step (6 × 10^6^) of each of the 200 simulations. The maximum usually is 1,200, because every protein occurs six times per simulation, except CenpA (once) and CenpN (two times). In the graphic presented here, the relative frequency of every possible binding is depicted. The histogram shows that all reactions were often realized, except Mps1-Hec1 and Zwint-Mis12 (for details, see Section 3.2.3. Simulation time for all 200 experiments is 2–3 days, due to the fact that some of them can be calculated in parallel.

We ran 200 simulations of an unattached kinetochore model variant with 6 × 10^6^ time-steps. For demonstration purposes, we focus on two features: First, how often is a reaction taking place during the 200 simulations (Figure 13). Second, we analyze the structural properties of the mitotic kinetochore using dendrograms (Figure 10).

We have six copies of all protein (except CenpA and CenpN); therefore, we did record how many overall reactions take place during the 200 simulations (a maximum of 1,200 times for each protein complex, and a maximum of 200 and 400 times for CenpA and CenpN, respectively). We found that all reactions take place most of time, except two interactions, which are: Mps1-Hec1 and Zwint-Mis12 (Figure 13). It has been suggested that *in-vivo* interaction between Hec1 and Mps1 requires additional modifications [145]. We did not consider any additional modifications in our model, and we see the interaction of only about 10% of the maximal possible interactions (Figure 13). This is indeed interesting and consistent with the experimental results [145]. The reaction, Zwint-Mis12, is happening about 30% during overall simulations; this is due to the geometrical constraint, where Zwint is far a way from Mis12, which makes it difficult to interact during each simulation. Instead, Zwint binds more often to Knl1 [101], which is consistent to our model output.

**Figure 14.**
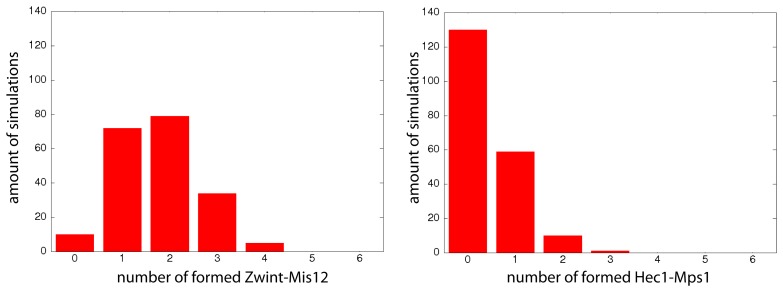
Analysis of the bindings Zwint-Mis12 and Hec1-Mps1. Studying Figure 13 reveals that two bonds (Zwint-Mis12 and Hec1-Mps1) are often absent. This bond formation can happen up to six times per simulation, because each of the proteins is present exactly six times. Therefore, it is interesting in how many simulations which number of these bonds is formed. These histograms show the distribution of the amount of the Zwint-Mis12 bonds (left) and of the Hec1-Mps1 bonds (right) that are realized during the 200 simulations. The X-axis refers to the number of realized bindings (Zwint-Mis12/Hec1-Mps1) in one simulation. The Y-axis refers to the amount of simulations. The Zwint-Mis12 bond it typically happening one to three times per simulation (two bonds are formed in 40% of the cases). The Hec1-Mps1 bond is not realized at all in 65% of the cases.

It is, however, not clear how many of each protein copy interacts during each simulation. To study this issue, we did record each single copy protein interaction during each simulation run. We show the result of the two typical examples for the Hec1-Mps1 and Zwint-Mis12 interaction (Figure 14). The distribution of the Hec1-Mps1 reaction of protein copies (Figure 14) shows that during the simulations, only a single copy (30%) interacts or barely a second copy (10%) of Mps1 molecules bind to Hec1 (Figure 14, right panel). Zwint-Mis12 has another behavior, where three protein copies make their reaction happen often (25%–40%).

It has been reported that the S-phase simulation model [56] has three families of structures, where over the simulations, always a single reaction was missing (CenpA/B, CenpB/W or CenpW/H3). We did similar structural analysis for our HKM mitosis model using both the RMSD and the paired RMSD distance metrics and generated a dendrogram using the UPGMA method (Un-weighted Pair Group Method with Arithmetic Mean) [57]. Our dendrogram (Figure 10) indicates that during mitosis, there is solely one family of structures. Thus, we support the idea that the mitotic kinetochore has one-family structures that are similar to each other. We justify the reason behind getting multiple structure for the S-phase model [56] as for the use of simple spheric molecular structures instead of considering detailed structural geometry for some important proteins and complexes. Hence, under the same geometry setting, a bridge between CenpA,-B, -W a H3 is impossible (the sum of the diameters is 7.5 nm, while the distance between the two nucleosomes is 14 nm).

Taken together, our model is consistent with the literature [145], supporting the idea that the interaction between Hec1 and Mps1 requires extra mechanism to take place. Additionally, our simulations suggest that the mitotic kinetochore has a single structure.

## 4. Discussion and Conclusions

In this study, we have presented, in a systematic, way how to apply a spatial rule-based approach to structural problems in molecular systems given interaction data and geometrical information. We have shown how various data sources can be integrated through a rule-based reaction network formalism and the specification of molecular geometries, including bond geometries. A molecule can be represented at different levels of detail. If nothing is known, molecules are represented as a sphere. Otherwise, arbitrary structures can be considered by structure templates (e.g., CenpC,-T,-Q and APC in our example).

**Figure 15.**
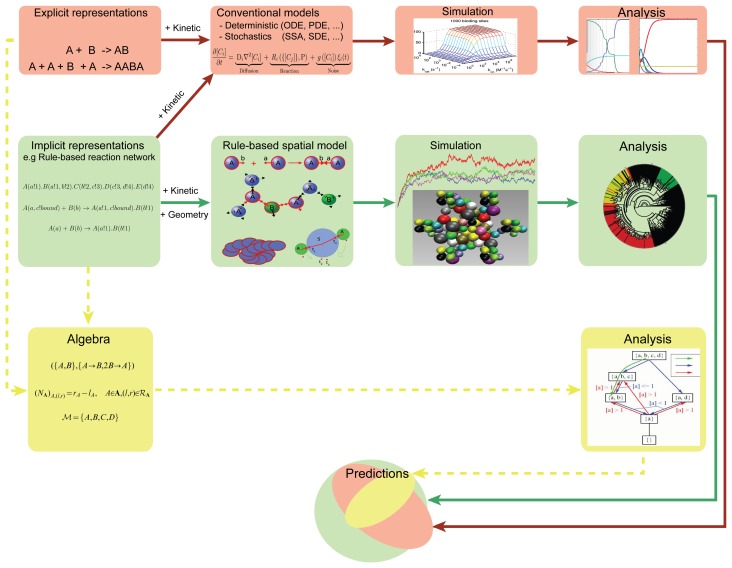
Illustration of modeling approaches in systems biology. Schematic representation summarizing our unconventional approach (in green), the classical or conventional approach (in red) and an outlook to future improvements (in yellow, dashed). ODE and PDE stand for ordinary and partial differential equation, respectively, whereby all other types of differential equations are implicitly included in this pathway, such as delay or fractional differential equation. SSA refers to the stochastic simulation algorithm and SDE refers to the general stochastic differential equation, which can be of any type (ordinary or partial).

One advantage of the spatial rule-based approach is a modular separation of the description of the reaction network from the molecule geometry (Figure 15). This means that the same description can be used for rule-based models that do not involve spatial properties.

### 4.1. Application Example

For demonstration, we applied our method to model the mitotic inner and outer kinetochore (HMK) for several variants of attached and unattached microtubules. Our analysis shows that a rosette-like chromatin structure can be assembled for both the unattached case or when a single microtubule is attached. In the case of two or three attached microtubules, we could not observe rosette-structures in simulation. With simulations of this process, we can obtain novel hints and inspirations for potential mechanisms. For example, in our simulation, microtubule attachment influences the rosette-shape, which might have a functional role additionally to the removal of SAC components. Furthermore, our model is consistent with the literature [145] supporting the idea that the interaction between Mps1 and Hec1 requires extra mechanism to take place.

### 4.2. Rule-Based Simulation Challenges

Even though our spatial, rule-based simulation approach solves some problems, many unsolved challenges remain: To begin with, there is a constraint on the systems that can be modeled with our current software implementation: at the moment, we cannot easily incorporate structural changes in proteins, e.g., a modification that would lead to different bond angles or different bond distances. While many systems, like the presented kinetochore model, can be modeled without these structural changes, we will include this feature in a future version by allowing a reaction that replaces one molecule with another, while keeping the bonds of this molecule intact.

Furthermore, from the modeling point of view, it would often be a great simplification if the BNGL language would allow the dynamic hierarchical definition of new *elementary molecules*, as proposed by Lemons *et al.* [146]. For example, a complex group of *elementary molecules*, like the CenpC,-T,-Q and APC from our example in Section 3, that is repeatedly referred to, might be defined as a new *elementary molecule*.

### 4.3. Analysis Challenges

After correctly setting up a meaningful simulation run, there are still many challenges ahead in understanding the simulation results: In addition to the analysis techniques presented in Section 2.7, many others are conceivable. For example, it might be sensible to reproduce experimental measurements, like FRET, FRAP or mutant studies, to have a more direct comparison between simulation and reality. On the other side, naturally for a rule-based reaction system, the same pattern definition that is used to specify the left-hand side of a reaction rule might be applied to count the number of molecules with a specific property in the reactor [13]. However, even simply determining the concentration of a specific chemical pattern poses an interesting problem: different counting schemes would lead to strongly differing numbers with different semantics. Imagine having a single long polymer in simulation and looking for the number of a “few-molecules-subset” pattern appearing in the reactor. Either the whole complex molecule could be counted once or any position fulfilling the pattern requirements could be counted or only non-overlapping patterns could be counted. Even counting non-overlapping patterns is not unique, because the outcome can depend on where the first pattern was placed.

Furthermore, the methods presented in Section 2.7 can be used as building blocks of more complex experimental setups. Due to their numeric nature, these setups can be automatized to a high degree. This allows experimental procedures that involve an—by human standards—intractable number of parameter sets. For example, one could use the paired RMSD to show that a number of models produce qualitatively the same structure. By iteratively removing reactions that do not alter the structure beyond some threshold, one can derive minimal models that yield a certain structure. Alternatively, one could define a meta-model of possible reactions and combine reactions known from wet-lab experiments with randomly chosen ones to automatically discover the model that is closest to a known wild-type structure. By restricting the paired RMSD to a subset of the *elementary molecules*, one can apply this method even in case of partial structural knowledge.

### 4.4. Future Challenges

A future challenge for systems biology is to develop other approaches that should allow for the derivation of properties without the need of simulation (Figure 15, yellow path). This could be based on abstract interpretation [147–149], coarse-graining [150–153], chemical organization theory [33,154] or model checking [155–158]. An overview of how our method relates to classical modeling and novel algebraic methods is presented in Figure 15. The same rule-base reaction network specification can feed into different analysis and simulation pipelines (Figure 15 red, green and yellow).

Rule-based modeling is already a step into the direction of re-usable models. The same is true for the hierarchical models [146]. Nonetheless, often, models have to be built again from scratch, when other aspects should be included. Rebuilding a model or making many changes are time-consuming. Furthermore, they have a specific language (ODE, SBML, *etc.*) which is not easily understood by non-modelers. To avoid the problem of these specific languages, an alternative approach, which is based on knowledge (called knowledge-based), could be employed. Our rule-based modeling in space approach serves as a first step towards a knowledge-based approach, since tables that include biological information are the input to this approach, where changes can easily be made. A future step would be to make the input to the rule-based models simpler by including biological sentences and searching for the missing information from databases and validating the model with mutations from these databases. Challenges in multi-scale modeling are not limited to systems biology, but also in many other fields, like in chemistry, social science, political systems and economics (e.g., [159–162]).

## Supplementary Materials

1. **Video 1:** The unattached kinetochore model.
2. **Video 2:** The attached kinetochore to single microtubule model.
3. **Video 3:** The attached kinetochore to three microtubules model.
4. **Video 4:** The attached kinetochore to two microtubules model.
5. **BioNetGen language (BNGL) sources files:** An example of complete sources files for the unattached kinetochore model variant (Video 1 has been generated with these codes).

Supplementary File 1 Supplementary (ZIP, 20783 KB)
